# The involvement of medical doctors in hospital governance and implications for quality management: a quick scan in 19 and an in depth study in 7 OECD countries

**DOI:** 10.1186/s12913-016-1396-4

**Published:** 2016-05-24

**Authors:** A. M. Rotar, D. Botje, N. S. Klazinga, K. M. Lombarts, O. Groene, R. Sunol, T. Plochg

**Affiliations:** Department of Public Health, Academic Medical Center, University of Amsterdam, Meibergdreef 9, 1100 DE Amsterdam, The Netherlands; Berenschot BV, Europalaan 40, 3526 KS Utrecht, The Netherlands; Professional Performance research group, Center for Evidence-Based Education, Academic Medical Center, University of Amsterdam, Amsterdam, The Netherlands; Faculty of Public Health and Policy, London School of Hygiene & Tropical Medicine, London, UK; Optimedis AG, Hamburg, Germany; Avedis Donabedian Research Institute (FAD), Universitat Autonoma de Barcelona, Barcelona, Spain

**Keywords:** Doctors in management, Quality management, Hospital governance, European comparison, Professionalism

## Abstract

**Background:**

Hospital governance is broadening its orientation from cost and production controls towards ‘improving performance on clinical outcomes’. Given this new focus one might assume that doctors are drawn into hospital management across OECD countries. Hospital performance in terms of patient health, quality of care and efficiency outcomes is supposed to benefit from their involvement. However, international comparative evidence supporting this idea is limited. Just a few studies indicate that there may be a positive relationship between medical doctors being part of hospital boards, and overall hospital performance. More importantly, the assumed relationship between these so-called doctor managers and hospital performance has remained a ‘black-box’ thus far. However, there is an increasing literature on the implementation of quality management systems in hospitals and their relation with improved performance. It seems therefore fair to assume that the relation between the involvement of doctors in hospital management and improved hospital performance is partly mediated via quality management systems. The threefold aim of this paper is to 1) perform a quick scan of the current situation with regard to doctor managers in hospital management in 19 OECD countries, 2) explore the phenomenon of doctor managers in depth in 7 OECD countries, and 3) investigate whether doctor involvement in hospital management is associated with more advanced implementation of quality management systems.

**Methods:**

This study draws both on a quick scan amongst country coordinators in OECD’s Health Care Quality Indicator program, and on the DUQuE project which focused on the implementation of quality management systems in European hospitals.

**Results:**

This paper reports two main findings. First, medical doctors fulfil a broad scope of managerial roles at departmental and hospital level but only partly accompanied by formal decision making responsibilities. Second, doctor managers having more formal decision making responsibilities in strategic hospital management areas is positively associated with the level of implementation of quality management systems.

**Conclusions:**

Our findings suggest that doctors are increasingly involved in hospital management in OECD countries, and that this may lead to better implemented quality management systems, when doctors take up managerial roles and are involved in strategic management decision making.

## Background

Hospitals are under increasing scrutiny to improve their performance. This does not only include the performance in terms of efficiency, but also, and increasingly, the performance in terms of quality and patient outcomes [1, 2]. Consequently, hospital governance is broadening its orientation from ‘control’ of costs and production towards ‘performance’ in terms of clinical outcomes. Hospital governance can be understood as a broad and ambiguous term. It essentially refers to the complex patterns of hospital-related decision-making at different levels shaping the actual “governance structure” for hospitals. It should be distinguished from hospital management which is narrower focusing on the day-to-day operational management of staff and services inside the hospital organization [2].

Against the backdrop of the so-called outcome-based hospital governance, doctors are drawn into hospital governance around the world [3, 4]. The argument is twofold. First, hospitals would improve their performance successfully, if led by people who are the key producers of clinical care. This seems common sense, as one needs a deeper understanding of the primary processes of patient care, i.e., professional expertise, for being able to improve the quality of care and patient outcomes for the better. The implementation of Quality Management Systems is considered one of the main mechanisms to realize performance improvement [5].

Second, hospital governance is notoriously strained due to its dual organizational structure - that is the co-existence of both managerial and professional decision making structures, as initially recognized and depicted in the concept of the ‘professional bureaucracy’ [6]. Later the US sociologist Eliot Freidson (2001) theorized that clinicians, being professionals, predominantly organize their practices following the logic of professionalization, while hospital managers follow the logic of management science or bureaucracy [7]. As both logics are often at odds with each other, tensional relationships between clinicians and hospital managers are common. Drawing doctors into hospital management would therefore ease the tensional relationship, and ultimately enhance the performance of the hospital [8–10]. More recent conceptualizations go beyond this dualism by emphasizing the intermingling of managerial and professional roles that currently seems to take place, especially when it is related to quality management [11, 12].

Studying the phenomenon of so called doctor managers fits in with the broader research on hospital governance. Research shows that there seems to be a convergent trend towards more independent governance models for hospitals in many countries as the traditional command-and-control model will no longer be viable [2, 13]. Hospitals will need to be governed as part of a network of outpatient and inpatient care providers that are concerned with patient responsiveness and better attention to the role of professionals. However, the comparative research on the impact of these newly emerging models is scattered and inconclusive. There are still many aspects that are not well understood.

Amongst others, few studies have investigated the relationship between hospital governance and quality performance. There are some studies reporting associations between quality performance and a range of initiatives including strategic goal setting for quality improvement, putting quality performance on the agenda of board meetings, monitoring quality dashboards, and appointing a quality committee [14–17]. Furthermore, the engagement of CEOs in quality management was associated with the success of quality improvement projects [18]. However, most studies on hospital governance have been carried out in the US, and the underlying mechanisms remain unclear.

Even more scarce is the research about the role of doctor managers and their impact on hospital performance. Just one study found on the basis of a secondary analysis of publicly available data of hospital trusts in the UK that those hospitals headed up by a doctor perform better than those led by lay managers [19]. Another cross-sectional study reported strong associations between the top-100 US hospitals media-generated ranking of quality and the medical background of the chief executive officer [20].

In conclusion, the processes through which doctor managers may mediate better hospital performance in terms of patient, quality and financial outcomes remains a ‘black-box’ thus far. Research on the relationship between doctor managers and quality of care is scarce, and international comparative research in this area is lacking.

Therefore, it was timely and relevant to provide a quick scan of the current situation with regard to doctor managers in the hospital systems of 19 OECD countries. In addition, we explored in depth this phenomenon as related to the implementation of Quality Management Systems in 7 OECD countries drawing upon the DUQuE project (“Deepening our understanding of quality improvement in Europe”) [21, 22]. Within this in depth study we explored the hospital governance and organisational context, the formal managerial roles doctor managers have taken up in their respective hospitals and we tested the association between the level of their involvement in hospital management and the level of implementation of Quality Management Systems.

## Methods

To address the research objectives, we combined empirical data from: 1) a quick scan drawing upon a short structured questionnaire amongst 19 country experts in the OECD’s Health Care Quality Indicator program, and 2) from a cross-sectional multilevel study, both on doctors’ involvement in hospital management and on the implementation of quality management systems, in the 7 OECD countries included in the DUQuE study.

### The impact of doctor managers in 7 OECD countries

For developing a quick overview of doctors’ involvement in hospital governance decisions in 19 OECD countries, we developed a short structured questionnaire. It was designed in four parts covering the (i) medical doctors’ involvement in governance decisions, (ii) the involvement of the governing board in clinical quality, (iii) use of quality indicators and (iv) suggestions for further country information sources.

The questionnaire was used to collect data through face-to-face interviews with OECD country experts attending the OECD Health Care Quality Indicators meeting in Paris, November 2013. These country experts were highly placed national public servants representing their national Ministries of Health.

Out of all participating country experts, 19 of them agreed to participate. Respondents were conveniently selected on their availability, but also purposefully selected to get all country experts from the seven countries included in DUQuE study (i.e., Czech Republic, France, Germany, Poland, Portugal, Spain and Turkey [21, 22]). All approached experts agreed to participate.

The data from the interviews were complemented with the literature to map the hospital governance and organisational context of doctor managers in the 7 OECD countries included in the DUQuE study in more detail. This was done against the comparative framework developed by Kirkpatrick et al. [23]. The framework distinguishes four key areas: strategic (governance), middle management, the nature of authority structures and development of nonclinical management roles [23].

### Cross-sectional data from the Duque project

The DUQuE project encompassed a multi-method, cross-sectional study design to collect quality-related information from European hospitals between May 2011 and February 2012. Included countries represented a geographical reach of the EU and various approaches to organizing health care. Czech Republic, France, Germany, Poland, Portugal, Spain and Turkey agreed to participate. These heath care systems provided a variation in the ratio between public and private hospitals, the number of teaching hospitals and hospital size. From each country, a random sample of 30 hospitals was recruited. The inclusion criteria were size (>130 beds) and treatment typology (acute myocardial infarction (AMI), hip fracture, stroke and deliveries) [22].

To address the main objective of studying the effectiveness of quality improvement systems in European hospitals, the DUQuE project team conceptualized, adapted and operationalized several constructs that were considered to be relevant to the quality of hospital care, including the doctors’ involvement in hospital management. This was done to capture a factor that could influence the uptake and implementation of quality improvement activities in hospitals.

#### Study population

In each participating hospital we sent questionnaires to 10 leading medical doctors. We defined a leading medical doctor as one who has a formal or informal leading role within the hospital. The identified leading medical doctors could indicate to what extent they had a formal management role and were involved in hospital management decision-making. In addition, the quality manager of each hospital was asked to fill in a questionnaire describing the level of implementation of their quality management system.

#### Measures used

The formal management role was measured by asking the respondents whether they held a formal management role and at what level (departmental or hospital). Answering categories to three items were 1 = Yes; 2 = No. In addition, we used a validated measure for professional involvement within hospitals [24]. This measure focused on the self-reported participation in hospital management decision-making by leading doctors and nurses. The professional involvement was measured in various areas of hospital management as perceived by leading medical doctors. Answering categories were: 1 = No involvement; 2 = Giving an opinion; 3 = Shared decision-making; 4 = Final decision-making responsibility. For the purpose of this study, we only used the self-reported formal management role and the perceived participation of leading medical doctors in hospital decision-making. The questionnaire is available on request.

The extent to which a quality management system is implemented, was measured by the Quality Management System Index (QMSI). This measurement tool was developed based on previous research by the DUQUE team and following a systematic review of the literature on conceptual models and measurement instruments [25]. QMSI measures the implementation of nine dimensions trough 46 items: quality policy documents, quality monitoring by the board, training of professionals, formal protocols for infection control, formal protocols for medication and patient handling, analysing performance of care processes, analysing performance of care professionals, analysing feedback patient experiences and evaluate results. Quality managers were asked to answer questions related to these dimensions. The implementation of the management system is expressed as an index (0–27), based on the extent of implementation of QI activities. Thus, the maturity index tells us something about how mature/well implemented the activity is within the hospital. In other words, a high score would mean that i) a wide scope of QI dimensions is addressed in the QM system, and ii) these dimensions are not only addressed at the level of strategic planning, but are actually implemented and their implementation is assessed (items are phrased based on the PDCA cycle).

#### Statistical analyses

Descriptive statistics were calculated to describe hospital characteristics (teaching status, ownership, and size) and demographic characteristics of participating leading medical doctors (gender, age, and number of years in job). Descriptive statistics are also reported for the outcome QMSI and main predictor (professional involvement in hospital management) in the analysis.

We analysed the relationship between professional involvement with the implementation of quality management as measured by QMSI. A multivariable linear regression model with random intercept by country was used to analyse the relationship between professional involvement (predictor) and the implementation of quality management systems as measured by QMSI (outcome). A linear regression model was applied to estimate the relationship between scalar predictor and outcome variables. We used a multivariable model to be able to adjust for hospital confounders. Hospital teaching status, ownership type and number of beds were treated as confounder in the model. Since we also expected that country differences can influence the abovementioned relationship, we included a random intercept for country in order to account for clustering of hospitals within countries. To determine statistical differences, the level of significance was set at 5 %. All statistical analyses were carried out in STATA.

## Results

### Doctors’ involvement in hospital management in 19 OECD countries

Table 1 provides the overview of the involvement of doctors in hospital governance within the 19 OECD countries. According to the OECD country experts, in 18 out of 19 countries medical doctors are part of the hospital top structure, and in some countries together with a ‘manager from another background, i.e., economics, health care administration, nursing, law or epidemiology.

**Table 1:** The phenomenon of doctor managers in 19 OECD countries

Country	MDs in Top Management Team	Formalized interaction between MDs and TM	Formalization into force since	MDs involvement in TM tasks	Type of tasks	MDs role in TM decisions
Belgium	Medical doctors	YES	1987	YES	mostly advisory	Consultative
	Economists					
	Managers					
	Nurses					
	Jurist					
Czech Republic	Medical doctors	YES	2012	YES	all - most managers are MD	Decisional
	Managers					
Denmark	Medical doctors	YES	1990	YES	support the development of clinical indicators & practice guidelines; education; human resources;	Decisional
	Economists					
	Managers					
	Nurses					
England	Economists	NO	N/A	YES	depending on internal processes and regulation; nothing standardized	
	Finance					
France	Medical doctors	YES	-	YES	e.g. infection management	Decisional (in practice)
	Managers					Consultative (in theory)
Germany	Medical doctors	YES	-	NO	N/A	Consultative
	Economists					
	Managers					
	Academia - where the case					
Israel	Medical doctors	YES	2009	YES	National Programme of Quality Indicators	Consultative
	Economists					
Italy	Medical doctors	NO	N/A	YES	only at a medical unit level	Consultative
	Managers					
Luxemburg	Medical doctors	YES	1998	YES	coordination of medical interdepartmental activities	Consultative
	Economists					
	Nurses					
Poland	Medical doctors	YES	1998	YES	advisory and decisional	Decisional
	Managers					Consultative
Portugal	Medical doctors	YES	many years ago	YES	e.g. infection control	Decisional
	Economists					
	Managers					
	Nurses					Consultative
	Legal					
Slovenia	Medical doctors	NO	N/A	YES	e.g. delivery of services, volume of services, waiting times;	Consultative
	Economists					
Spain	Medical doctors	YES - depends on the region	2006	YES	local guidelines	Decisional
	Managers					
Sweden	Medical doctors	NO - in most regions	N/A	YES	e.g. setting Quality Indicators and guidelines	Consultative
	Managers					
Turkey	Medical doctors	NO	N/A	YES	all hospital management task	Decisional
	Economists					
Japan	Medical doctors	YES	2000	YES	CEO	Decisional
Singapore	Medical doctors	YES	2009	YES	almost all hospital management task	Decisional
	Managers					
South Korea	Medical doctors	YES - only in large hospitals	2000	YES	e.g. most decisions on hi-tech acquisitions and quality assessment	Decisional
	Managers - rarely					
Canada	Medical doctors	YES	2003–2008	YES	almost all hospital management task	Consultative
	Economists					
	Managers					
	Epidemiologists					

Fourteen country experts reported that the hospitals in their countries have adopted a formalized structure based on mutual responsibilities, working guidelines and policies that regulates the relationship and interaction between the medical doctors and governing board. These formalized interactions are reported to exist since the late 80s’ and countries have adopted them gradually over time. The experts reported that medical doctors are involved in hospital governance in 18 countries (all except Germany). The types of tasks in which medical doctors are involved create a mixed picture. In 9 of the countries the involvement is clinically related limited to medical activities while in another 8 countries the role is broader including all other managerial activities (e.g. budget holder, human resources etc.). Amongst the clinically related activities, the main reported focus is related to guideline development and quality indicators (e.g. Denmark, Israel, Spain). The medical doctors involved have the role to oversee the delivery and volume of services, waiting times (Slovenia) and to take decisions on hi-tech acquisitions (South Korea).

The role of the medical doctors in hospital governance decisions is formalized through a set of working guidelines in all countries except England. It was reported that in 8 countries the role is consultative, in 7 countries decisional and in the remaining, the role is both consultative and decisional.

### Doctor managers in 7 OECD countries

#### Sample characteristics

There were 188 hospitals participating in the Duque study (Table 2). Most hospitals were public (*n* = 156, 82.9 %), and medium sized with 200 to 500 beds (*N* = 79, 42.0 %). Almost half of the hospitals had a teaching profile (*N* = 81, 43.0 %). In 188 hospitals there were 1670 leading medical doctors who completed the questionnaire, yielding an overall response rate of 88 %. Questionnaire completeness was high; 1505 respondents (90 %) responded to all questions in the survey.

**Table 2:** Characteristics of the DUQUE study sample

Characteristics of the hospitals	N	%
All Hospitals	188	(100)
Teaching Hospitals	81	(43.0)
Public Hospitals	156	(82.9)
Approximate number of beds in hospital	N	%
<200	18	(9.5)
200–500	79	(42.0)
501–1000	62	(32.9)
>1000	29	(15.4)
Characteristics of the leading doctors^a^		
Gender	N	%
Male	1151	(68.9)
Female	510	(30.5)
Gender missing	9	(0.5)
Age (years), Mean (SD)	49.3	(8.3)
Number of years since completion of professional training, Mean (SD)	21.6	(9.9)
0–5 years, N (%)	106	(6.3)
6–10 years, N (%)	139	(8.3)
11–20 years, N (%)	483	(28.9)
21+ years, N (%)	914	(54.7)
Missing, N (%)	28	(1.6)
Member of professional society, N (%)	1464	(87.6)

^a^Includes attending physicians and residents-in-training

Most of the respondents were male (*N* = 1151, 68.9). Their average age was 49.3 (SD 8.3) years. All respondents were experienced, as reflected in the 21.9 (SD 9.7) years on average since they completed their professional training. Most of the respondents were member of a professional society (*N* = 1464, 87.6 %).

#### Hospital governance

Table 3 provides a more detailed description of the hospital governance and organisation within the 7 OECD countries included in the DUQuE study. The data fits in with the bigger picture that hospital governance practices move away from governing hospitals through parallel hierarchies, with doctors represented by a senior medical committee, sometimes with powers to veto management decisions. All 7 countries are changing or have changed the hospital governance. The focus is now on strong medical management roles on departmental level and all doctors reporting through a single, unitary chain of command to a clinical director who in turn is accountable to the chief executive or general manager of a hospital. Notwithstanding the convergent trend, the hospital governance and organisation arrangements differ amongst the 7 OECD countries.

**Table 3:** Hospital governance and organization in 7 OECD countries

Country	Hospital governance	Middle tier	Management authority	Development of non-clinical management
Czech Republic [27]	Hospital governance varies depending on the type of hospital. State hospitals usually have simple organisational structures headed by a director controlled by a given state department. Municipal hospitals are governed by boards of directors consisting of between six and ten employees (mainly medical doctors). Privately owned hospitals have top-down traditional pyramid ownership structures, where a board of directors consists of company managers and a supervisory board represents owners and operate under standard profit oriented business principles.	At department level, chiefs of ward/clinic are the important medical management roles.	Interests of board members are usually quite distinct from each other and therefore corporate performance is not always regarded as the most important objective. Accordingly, disputes among the different in-groups result in higher autonomy of management at the expense of owners.	Approximately half of the hospitals have now a senior manager with a non- medical qualification.
France [28]	The governance of hospitals is characterised by a tripartite structure comprising 1) an executive council (including the General Director and President of the Medical Council); 2) an administrative (or supervisory) council representing external stakeholders, and 3) a medical council (or commission) representing medical doctors.	After 2007 hospitals are organised into clinical and non-clinical activity *poles* run by a triumvirate of director, administrative manager and nurse manager. All *poles* also have a council with strong representation from medical heads of clinics. In theory activity poles operate as semi-independent businesses contracted by the hospital.	Despite efforts to streamline management, authority at hospital and activity pole level remains fragmented, characterised by internal checks and balances.	Public hospitals employ non-clinical managers both centrally and, increasingly, within at activity pole centres. A large proportion of these ‘managers’ are also civil servants (with legal or political science backgrounds) with statutory roles.
Germany [29]	Hospitals have an executive committee and sometimes an advisory committee composed of senior medical doctors. Traditionally the executive committee is composed of a ‘troika’ consisting of as a medical director, the top representative of nurses, and the head of the hospital’s administration.	At the level of departments doctors may design clinical management filling in a regulatory gap that results from the absence of a comprehensive troika structure. New modes of performance management are established, but accountability structures are not adequately adapted. This creates space for strategic action of medical doctors as the most powerful group in hospital governance.	Hospital authority remains varied, with a lot of discretion given to owners and boards. Today’s hospitals may either organize their medical departments as self-dependent hospitals “sub-enterprises,” with a head doctor (clinical director) holding a more encompassing budget responsibility, or maintain head physicians as mere medical experts and directors of a more centrally controlled medical department.	In the hospitals CEO positions above the troika are established and often fulfilled by a non-clinical manager.
Poland [30]	Most hospitals are public owned by local governments and universities. Around 30 per cent of them have been transformed into “non-public” entities, operating under the same legal framework as commercial companies. Hospitals have an executive board composed of a CEO and a medical director.	At department level, chiefs of ward/clinic are the important medical management roles. The post is held by a senior doctor, reporting to a medical director, who is responsible for all ward operations. Some, but not all, chiefs also have responsibility for the financial standing of the unit.	At departmental level the structure lacks coherence. Centralised managerial controls increasingly intervene in medical budgets, thus reducing medical power, while organisational controls are weaker and flexibility of doctors higher in the area of quality and safety management.	The CEO role is increasingly held by non-medical professionals.
Portugal [31, 32]	Troika structure at top level with some flexibility. Managers are in the lead of the board of directors. The administration board consists of medical doctors and nurses.	The Board appoints a director for each medical area (service or department) from among the most qualified doctors in the professional career.	In a situation of poorly established accountability structures, the ability of doctors to use management strategically is increasing on the level of departments and in the area of quality management.	Non-medical managers, who are now in control of the hospital board of directors, are expected to play a crucial role in hospital governance.
Spain	Troika structure relevant at all levels; but double structure of general & ‘doctors only’ boards assures flexibility & medical power.	Medicine is expanding into management and this is driven by both departmentalisation and the establishment of clinical management with doctors utilising control bottom-up.	Top-down with some bottom-up controls; troika structure expanding, but quality mainly managed by medical doctors; weak coordination & flexibility.	There are non-medical managers in the system.
Turkey [33, 34]	The management of public hospitals is under government control.	At the departmental level medical doctors have to work as managers in a strong top down hierarchy.	Strong hierarchy with integration of medical power.	There are few qualified health managers who took part in a management course.
	The chief medical doctor is the manager of the hospital and is assisted by hospital managers responsible for administrative, financial and technical issues and by chief nurses responsible for nursing services. Under the ‘pilot hospital autonomy law’ (2007) the traditional centralised governance structure is replaced by a ‘public-enterprise’ model whereby hospitals joining the pilot project would be managed by boards, while remaining affiliated to the Ministry of Health.			

#### Formal management roles

A small proportion of our respondent sample (9.8 %) held no formal management role (Table 4). More than half of the respondents (51.7 %) held a formal management role at the department level. The rest held formal management roles at the hospital level only (5.3 %) or both at the hospital and department levels (17.5 %). When it comes to the professional involvement in hospital management decision-making, the majority of the respondents only gives their opinion. This did not differ much across the four decision-making areas. Only when it comes to the managing of medical practice, the respondents share decision-making responsibility (mean 2.8; SD 0.7).

**Table 4:** Doctors’ involvement in hospital management in 7 European countries

Having a formal management role	N	%
No formal management role	165	(9.8)
Formal management role at the department level only	864	(51.7)
Formal management role at the hospital level only	90	(5.3)
Formal management role at both the department and hospital level	293	(17.5)
Formal management role missing/unknown	258	(15.4)
Professional involvement in management	Mean	SD
* “How would you describe your participation within the following decision-making areas?”* * 1 = No involvement, 2 = Giving an opinion, 3 = Shared decision making, 4 = Final decision making responsibility*		
Administration and budgeting	2.1	0.7
Managing medical practice	2.8	0.7
Strategic management	1.7	0.6
Managing nursing practice	1.8	0.6

#### Professional involvement and quality management systems

Associational analysis between the professional involvement measure and the quality management system index (QMSI) showed a significant positive association between the professional involvement in strategic management and the QMSI. This means that those hospitals where leading medical doctors share decision-making or have final decision making responsibility in strategic issues have a higher score on QMSI (Table 5).

**Table 5:** Regression coefficients for the association between professional involvement in management (4 subscales) and hospital quality management indices QMSI

	QMSI^a^ (mi)
	*b* (SE)
Strategic management	3.86 (1.61)*
Administration and budgeting	1.37 (0.96)
Managing medical practice	2.57 (1.52)
Managing nursing practice	0.35 (1.12)
Professional involvement scale (total)	0.68 (0.34)*

^a^For each of the 4 scales of professional involvement in management, we estimated its association with QMSI using a multivariable linear regression model with random intercept by country, adjusted for confounders at hospital level (number of beds, ownership, teaching status)

*Significance *p* < 0.05

## Discussion

We were able to conduct an international study on the involvement of leading medical doctors in hospital governance in OECD countries, most in depth in 7 European countries. The large sample of 1670 respondents in 188 hospitals yielding a response rate of 88 % provides novel evidence in this research area that is still in its infancy.

Hospitals are broadening their focus from steering on efficiency, productivity and cost control towards steering on improvement of outcomes in terms of quality and patient outcomes [1, 2]. Furthermore, indicative evidence shows that involving doctors in hospital governance is positively associated with the performance of hospitals [19, 20]. The findings of this study confirm that involving of medical doctors in hospital management is common practice in almost all of the 19 OECD countries on both departmental (middle management) and hospital level. This finding is quantitatively substantiated by our in depth study in 7 OECD countries showing that almost all leading medical doctors have taken up formal management roles, mostly at the departmental level, but also on hospital level or both. Still, 1 out of 10 leading medical doctors in our study reported not to hold any formal management role. Thus, being a leading medical doctors does not automatically go along with a formal managerial position.

Moreover, the formalisation of managerial roles is not automatically accompanied by final decision-making responsibility. On average most medical doctors are consulted and asked to give their opinion. Thus, it seems that doctors execute management tasks, but without the mandate to really take the managerial decisions. This is in line with the contextual data on the hospital governance and organisation within these 7 countries sketching an ambiguous role of leading medical doctors in hospital management, which is also highlighted in the literature [11, 12].

Furthermore, our findings indicate that leading medical doctors actually having decision-making responsibility in strategic management issues matters. It is associated with higher levels of implemented quality management systems. This finding supports the hypothesis that involving medical doctors in hospital management leads to better performing hospitals mediated through the implementation of quality systems. Moreover, it sheds light on *why* drawing medical doctors in management is associated with better hospital performance [19]. In the larger Duque study a clear relation was found between the implementation of departmental quality strategies and clinical practice [26]. Hence a relation with the medical management roles on departmental level is a fair assumption. Our finding also suggest that the actual decision making responsibility in strategic management areas is critical for the assumed relationship with hospital performance. Thus, a critical factor may not be the uptake of managerial tasks by doctors in itself, but giving them also the actual decision-making responsibility in strategic management areas.

### Strengths and limitations of the study

This study has a number of limitations that need to be highlighted. First, we cannot draw conclusions on causality due to the cross-sectional nature of both the quick scan and the Duque study. Second, international research on the involvement of medical doctors in hospital governance needs to account for contextual differences across countries. Hospital governance systems differ significantly amongst OECD countries [2]. This also influences how medical doctors take up managerial roles and how quality systems are implemented. For this reason, we complemented the DUQuE study with a quick scan yielding contextual data on doctor managers in 19 OECD countries. Within the DUQuE study, we tackled this issue by specifically developing and validating an instrument that fitted our research objective [24]. Above all, we were able to adjust for different country and hospitals characteristics in ways that allowed us to address competing explanations and plausible (non)causal associations, while minimizing sources of bias [22]. A third limitation is related to the sampling strategies employed. The convenient sampling of country experts for the quick scan may have led to some bias. Even so, the random sampling of the countries, hospitals and medical doctors for the Duque study may have led to selection bias (e.g., self-selection by the hospitals and medical doctors accepting to participate). Therefore, generalization to participating countries and hospitals was limited.

## Conclusions

Our findings suggest that in OECD countries medical doctors are increasingly involved in hospital governance on both departmental (middle management) and strategic hospital level. Most importantly, doctors involvement is associated with better implemented quality management systems, especially when doctors are involved in strategic management decision making. Hence increased focus on hospital performance seems to go along with strong medical involvement in hospital governance.

## Ethics approval and consent to participate

Not applicable

## Consent for publication

Not applicable
